# Current evidence on pre-swallowing tasks during FEES: are they predictive of swallowing function?

**DOI:** 10.1016/j.bjorl.2023.101280

**Published:** 2023-06-12

**Authors:** José Vergara, Anna Miles

**Affiliations:** Department of Surgery, Head and Neck Surgery, University of Campinas, R. Tessália Vieira de Camargo, 126, Campinas 13083-887, SP, Brazil; Department of Speech Science, School of Psychology, University of Auckland, Auckland, New Zealand

Thirty-five years ago, the flexible endoscopic evaluation of swallowing (FEES) was described for the first time. FEES is a gold standard instrumental assessment of oropharyngeal swallowing function using flexible laryngoscopy. FEES usually includes a part 1, in which the anatomy of the pharynx/larynx is observed and movements of structures are evaluated through motor tasks. These often include the pharyngeal squeeze maneuver (sustained/pressed phonation “eee”), velopharyngeal closure (“puh-puh,” “fifty-fifty,” or swallow), base-of-tongue retraction (words with postvocalic /l/, e.g, “Paul is tall”), glottic closure/vocal fold and arytenoid mobility (assessed during coughing or sustained breathing, sustained “ee”, repeated “ee-ee-ee”, and pitch glides); as well as the observation of epiglottic inversion during a dry swallow. These tasks allow the clinician to build their working hypotheses regarding intact and problematic components of the pharyngeal phase of swallowing i.e. pharyngeal constriction for bolus clearance and vocal fold closure for airway protection. Voice and swallowing share neural and musculoskeletal substrates. Yet, the strength of evidence for motor and speech tasks predicting swallowing function is variable making it hard for clinicians to confidently build working hypotheses during part 1 of their FEES. This results in some clinicians leaving this part of their FEES out and missing valuable clinical information about their patient. What is the evidence for these motor speech tasks components of the FEES procedure?

## Pharynx

One of the most validated motor tasks, with the greatest potential to predict swallowing function, is the pharyngeal squeeze maneuver (PSM), which is considered a substitute measure of pharyngeal strength during FEES. Recently published, Miles and Hunting report on 222 inpatients referred for FEES and demonstrated a correlation between PSM and pharyngeal constriction during videofluoroscopy (in those who had both assessments) and a strong association between PSM and accumulated secretions, aspiration, residue and diet on discharge from hospital.^1^ PSM also correlated with other motor functions including vocal fold immobility, reduced cough peak flow and reduced swallow frequency.^1^

## Velopharyngeal closure

Recently, a modification of PSM, called the Velopharyngeal Squeeze Maneuver (VPSM), has been studied in patients with dysphagia.^2^ The researchers demonstrated that the absent VPSM is able to predict the presence of aspiration. Patients with absent VPSM also had severe impairment in pharyngeal contraction. The authors report that this was the first study to demonstrate the clinical utility of observing velopharyngeal closure during a speech task and its relationship to functional swallowing.^2^

## Vocal fold function

Glottic closure is essential for airway protection during swallowing and coughing, thus, dysphagia/aspiration often occurs in patients with vocal fold paralysis, with an estimated prevalence of 55%–69%.^3^ The relationship between aspiration and vocal fold paralysis is well documented.^3^ While vocal fold adduction is an essential component of airway closure for swallowing, we believe that the clinical utility of these motor tasks may be improved by assessing other components of airway closure including epiglottic inversion and arytenoid mobility. This could provide further information about laryngeal valve closure during swallowing, an event not visible during FEES.

## Hyoid and laryngeal displacement

Recently, a study by Jijakli et al. evaluated the association between epiglottic inversion (observed during FEES) with the biomechanical events of swallowing (observed during videofluoroscopy) in a small cohort of 25 patients with dysphagia. The researchers wanted to know whether reduced hyoid and laryngeal excursion on videofluoroscopy would be associated with absent epiglottic inversion on FEES. Patients were divided into three groups: complete, reduced, and absent epiglottic inversion. The study demonstrated that the reduction of base-of-tongue retraction, reduction of hyoid excursion, reduction of laryngeal elevation and reduction of pharyngeal constriction contribute to the decrease of epiglottic inversion observed in FEES. However, the assessment of epiglottic inversion was unable to discriminate between patients with normal versus impaired hyolaryngeal elevation. This small pilot study perhaps suggests that observations of epiglottic inversion should be interpreted with caution, and cannot infer the specific movement of the hyolaryngeal complex.^4^

## Base-of-tongue retraction

Base-of-tongue retraction, while often included in a FEES protocol, has little scientific evidence to relate task performance to swallowing function. Langmore et al. studied the relationship between pre-swallowing tasks assessed during the part 1 of the FEES (including base-of-tongue retraction) and functional swallowing outcomes in patients with post-extubation dysphagia. The only task that showed a significant relationship with aspiration was decreased pharyngeal squeeze. No association was observed between base-of-tongue retraction and swallowing impairments in this specialized population.^5^ To our knowledge, the base-of-tongue retraction motor task has not been explored further.

## Clinical implications and future directions

Despite limitations in current evidence, researchers and clinicians recommend that pre-swallowing tasks are included during FEES (Representatives of the American Board of Swallowing and Swallowing Disorders; ASHA Special Interest Group 13: Swallowing and Swallowing Disorders - Dysphagia https://pubs.asha.org/doi/10.1044/2021_AJSLP-20-00348). While some motor tasks may not been researched extensively, these tasks assess swallowing related actions in isolation and support clinician diagnostic decision-making. Knowing there is a vocal fold paralysis or poor pharyngeal squeeze provides valuable information about how a patient may perform during oral trials, as well as what the underlying biomechanical cause of functional deficits during oral trials. A deficit observed during a motor or speech task that has little evidence may need to be interpreted with caution. For example, if a clinician observes absence of epiglottic inversion or base of tongue retraction during part 1 of their FEES procedure, they might pay focused attention to these structures during oral trials during the FEES and may recommend a videofluoroscopy study to further investigate pharyngeal function. It is also recommended that clinicians stay current and adapt their clinical practice as new evidence becomes available over time.

The pre-swallowing tasks assessed in FEES are simple, quick and low risk and provide valuable information about a patient’s likely performance during oral trials as well as their underlying biomechanical impairments. The clinical utility of assessing tongue base retraction, velopharyngeal closure and epiglottic inversion should be a priority on the research agenda. Researchers should not limit themselves to studying only predictive values and validity, but also reliability and reproducibility in clinical practice. The creation and validation of new tools with clear definitions that allow a visual classification (e.g., normal versus impaired) for all pre-swallowing tasks is warranted.

## Conflict of interest

The authors declare no conflicts of interest.

## Funding

No funding.
